# Factors assessed in the first year of a longitudinal study predict subsequent study visit compliance: the TEDDY study

**DOI:** 10.1186/s40001-023-01563-z

**Published:** 2023-12-15

**Authors:** Jessica Melin, Kristian F. Lynch, Markus Lundgren, Carin Andrén Aronsson, Helena Elding Larsson, Suzanne Bennett Johnson

**Affiliations:** 1 Department of Clinical Science, Lund University, CRC Hus 60 Pl 11, Box 50332, 202 13 Malmö, Sweden; 2 Health Informatics Institute, Morsani College of Medicine, University of South Florida, Tampa, FL USA; 3 Department of Pediatrics, Kristianstad Hospital, Kristianstad, Sweden; 4 Department of Pediatrics, Skåne University Hospital, Malmö, Sweden; 5 Department of Behavioral Sciences and Social Medicine, Florida State University College of Medicine, Tallahassee, FL USA

**Keywords:** Study visit compliance, Study satisfaction, Child, Parent, Longitudinal study, Type 1 diabetes

## Abstract

**Background:**

Compliance with a study protocol is central to meeting its research goals. In longitudinal research studies, data loss due to missed visits limit statistical power and introduce bias. The Environmental Determinants of Diabetes in the Young (TEDDY) study is a longitudinal multinational (US, Finland, Germany, and Sweden) investigation of children at risk for type 1 diabetes (T1D) that seeks to identify the environmental triggers of islet autoimmunity and T1D. The purpose of the current study was to identify sociodemographic variables and maternal characteristics assessed in the first year of TEDDY that were associated with study visit compliance in the subsequent 3 years.

**Methods:**

Sociodemographic variables, maternal life-style behaviors, post-partum depression, maternal reactions to the child’s T1D risk, and study-related variables were collected at child-age 6 months and 15 months. Multiple linear regression was used to examine the association of these variables to study visit compliance in the subsequent 3 years.

**Results:**

Study visit compliance was highest in Sweden (*p* > 0.001), in children who were their mother’s first child (*p* > 0.001), and whose mothers were older (*p* > 0.001) and more satisfied with the TEDDY study (*p* > 0.001). Father participation was also associated with better study visit compliance (*p* > 0.001). In contrast, children whose mothers smoked (*p* > 0.001), suffered from post-partum depression (*p* = 0.034), and were more anxious about their child’s T1D risk (*p* = 0.002), completed fewer visits. Father’s study satisfaction was also associated with study visit compliance (*p* = 0.029); however, it was not significant in models that included maternal study satisfaction.

**Conclusions:**

Sociodemographic variables, maternal characteristics—including study satisfaction—and fathers’ participation in the first year of a longitudinal study were associated with subsequent study visit compliance in a sample of children genetically at-risk for T1D followed for 4 years. This information can inform future strategies designed to improve study visit compliance in longitudinal pediatric studies.

*Trial registration:* NCT00279318, 06/09/2004.

**Supplementary Information:**

The online version contains supplementary material available at 10.1186/s40001-023-01563-z.

## Background

Compliance with a study protocol is central to meeting its research objectives. In longitudinal research studies, data loss due to missed visits can reduce power, introduce bias and can be particularly problematic when the timing of biological markers or other outcome variables is critical to the study’s goals. Longitudinal research with pediatric populations is challenging, since adherence to the study protocol depends both on the parents and the child. Although several studies have focused on factors associated with study drop out [1–6], identifying factors associated with study visit compliance among those who remain in longitudinal pediatric research studies is equally important. However, only a few pediatric trials have addressed this issue. A study of children with Marfan syndrome over a 3-year period found better visit compliance among younger patients, white vs African American patients, and those with no family history for the disease. In addition, there were important site differences; sites with higher study visit completion rates used more strategies, had more staff resources and lower staff turn-over [7]. The Childhood Asthma Management Program followed over 1000 children with asthma for 4–6 years. Older children with milder asthma and who had more behavioral, emotional, or social problems at study inception missed more visits over the course of this longitudinal study [4].

Type 1 diabetes (T1D) is one of the most common autoimmune diseases in children and is caused by destruction of the insulin producing beta-cells in the pancreas. Both the genetic profile associated with increased risk for this disease as well as the islet autoantibodies associated with this autoimmune process have been identified. While most children who are genetically at-risk for T1D will not go on to develop the disease, almost all children who develop multiple islet autoantibodies will develop the disease [8]. The Environmental Determinant of Diabetes in the Young (TEDDY) study is an international longitudinal investigation that seeks to identify the environmental triggers of islet autoimmunity and T1D by following children at increased genetic risk from birth to 15 years of age [9]. Study visit compliance is critical to the timely identification of islet autoantibodies in this population.

Previous TEDDY reports have identified factors associated with study drop-out that may be important for study visit compliance, including sociodemographic factors, maternal life style behaviors, maternal reactions to the child’s risk for T1D, and the satisfaction with TEDDY study participation; father participation was also associated with greater study retention [6, 10]. Compliance with Oral Glucose Tolerance Test protocol among multiple islet autoantibody positive children was also examined in TEDDY. Children from Sweden and Finland compared to children from US and Germany, children whose mothers were more satisfied with study participation, and who monitored their child at home by checking the child’s blood glucose were more likely to be adherent with the Oral Glucose Tolerance Test protocol. In contrast, children seen on a long-distance protocol (instead of attending a TEDDY study center), who had a first degree relative with T1D, and whose mother underestimated the child’s T1D risk were less compliant with this aspect of the protocol [11].

The purpose of this study was to identify sociodemographic and maternal characteristics assessed in the first year of the TEDDY study that predicted study visit compliance in the subsequent 3 years among families who remained enrolled in the study for at least 4 years. Potentially modifiable characteristics were of particular interest, since these might be used to improve study visit compliance in the future. Since maternal study satisfaction has been associated with both TEDDY drop-out and Oral Glucose Tolerance Test compliance [10, 11], this potentially modifiable variable was of particular interest. In TEDDY, study satisfaction has been associated with both site-related factors (country, staff consistency) and parental characteristics (accuracy of the parents’ perception of the child T1 D risk and beliefs that something can be done to prevent the disease in the child)[12].

Based on the literature and previous publications in TEDDY, we hypothesized that the following variables measured in year 1 of the TEDDY study would predict study visit compliance in years 2–4: (1) sociodemographic variables, including country of residence, child age, sex, first born status, ethnic-minority status, family history of T1D, and maternal age [6, 7, 10, 11]; (2) maternal life style behaviors, including smoking and working outside the home [6, 10]; (3) maternal post-partum depression [2]; (4) maternal reactions to the child’s risk for T1D, including her perception of the child’s risk for T1D and her anxiety about that risk [6, 10, 11], and (5) study-related variables, including parent study satisfaction and father participation in the TEDDY study [6, 10, 11]. To our knowledge this is the most comprehensive attempt to identify factors measured in year 1 of a longitudinal pediatric study that predict study visit compliance in the subsequent 3 years.

## Methods

### The TEDDY study

This multinational longitudinal study is following children at genetic risk for T1D from birth to 15 years of age in an effort to identify the environmental triggers of islet autoimmunity and T1D. Children were screened at birth for genetic risk for T1D using human leukocyte antigen **(**HLA) genotyping in four different countries (Finland, Germany, Sweden, and US) between 2004 and 2010 [13]. Over 8,600 HLA-eligible children were enrolled before 4.5 months of age and followed with four visits per year until 4 years of age and then two times per year until 15 years of age. The protocol includes interviews, questionnaires, food records, blood draws and other samples. Blood samples were collected at each visit and screened for three different islet autoantibodies: glutamic acid decarboxylase, insulinoma-associated protein 2, and insulin. The frequent study visits were designed to permit the investigators to identify the onset of islet autoantibodies in a timely manner. Parents of children who develop islet autoantibodies during the study were informed of their child’s increased risk for T1D. Each country’s ethical research review board approved the study and all parents’ consent to the study at enrollment [9].

### Study sample/population

For this study, the focus was on the still enrolled, never withdrawn, and active participants (with at least one visit per year) at child-age 48 months (*n* = 4916). We excluded children who developed any of the T1D-related autoantibodies before 4 years of age (*n* = 316), as this information may affect study participation and is the subject of a separate investigation. The current analysis consisted of 4600 TEDDY families who were active participants from 3.5 months to 48 months of age.

### Study visit compliance

To measure study compliance at 4 years of age, we used the number of completed visits between the child-age 18 months and the child-age 48-month visit; 11 visits were scheduled during this interval. Since the study inclusion criteria included at least one visit per year, the range of the number of completed visits was necessarily restricted from 3 to 11.

### Predictors of study visit compliance

#### Sociodemographic variables

Information about country of residence, child sex, mother’s age at the birth of the child, child ethnic minority status, and if the child belongs to a family with a relative (mother, father, or sibling) with T1D (yes/no) was collected at initial screening in the study. US participants were considered as ethnic minority if the mother was not born in the US, the mother’s first language is not English, or the child is Hispanic or identifies as a race other than white. For the European subjects, if the mother was born in another country, or her first language is other than the language of the country in which the child resides, the child is considered an ethnic minority. Additional information collected at the child-age 9-month visit included whether the child was the mother’s first child (yes/no), whether the mother is a single parent (yes/no) and the mother’s education level (graduated college/university or higher; graduated trade school or some collage/university education; primary school through some trade school).

#### Maternal lifestyle-related variables

At the child-age 9-month visit, information about whether the mother smoked (yes/no) and worked outside the home (yes/no) was collected.

#### Post-partum depression

At the child-age 6-month visit, mothers completed the Edinburg Postnatal Depression Scale. Mothers’ scores were coded as ≥ 13, the cutoff score for clinical depression (yes/no) [14].

#### Maternal reactions to the child’s T1D risk

Maternal anxiety about the child’s T1D risk, the accuracy of the mother’s perception of the child’s T1D risk, and the mother’s belief that something can be done to reduce their child’s T1D risk was assessed using a questionnaire, administered at the child-age 6 months and 15-month visits and annually thereafter. In the current analysis, we used data collected at the first annual (child-age 15 months) visit. If data were missing at the 15-month visit, data from the questionnaire completed at the child-age 6-month visit were used. Maternal anxiety about the child’s T1D risk is measured by a 6-item short form [15] of the state anxiety component of the State Trait Anxiety Inventory (STAI)^1^ [16]. The 6-item short form has been used to measure parental anxiety in numerous T1D screening studies [15, 17–19]. Parents are asked to respond to the following question: “When you think about your child’s risk for developing diabetes, you feel”: followed by three anxiety absent and three anxiety present items. The score from the short 6-item State Anxiety Inventory (SAI) was converted into the original 20-item score [15]. The SAI is reliable in this study population (*α* = 0.90 for the child-age 15-month questionnaire). The measure appears stable between the child-age 6- and 15-month assessments (*r* = 0.74).

The accuracy of the mother’s perception of her child’s T1D risk was measured by the following question: “Compared to other children, do you think your child’s risk for developing diabetes is: Much lower, somewhat lower, about the same, somewhat higher, or much higher.” Mothers responding much higher or somewhat higher were considered accurate, all others as inaccurate. This measure has been used in previous studies with parents of children at risk for T1D [11, 12, 18, 20].

A mother’s belief that something can be done to reduce the child’s T1D risk was measured by two questions. “I can do something to reduce my child’s risk of developing diabetes” and “Medical professionals can do something to reduce my child’s risk of developing diabetes.” On a five-point (0–4) scale, mothers agreed or disagreed with the two statements and answers were combined, with higher scores indicating stronger belief that something can be done to reduce the risk of the child to developing the disease. Reliability estimates for this study population ranged from *α* = 0.66 on the child-age 15-month questionnaire to *α* = 0.71 on the child-age 6-month questionnaire. The measure has been used in previous studies with parents of children at risk for T1D [11, 12, 21].

#### Study-related variables

At child-age 6 months, 15 months and annually thereafter a questionnaire was used to measure parent study satisfaction using three items: (1) “Overall, how do you feel about having your child participate in the TEDDY study? (scored 2 = like it a lot, 1 = like it a little, 0 = it is ok or dislike it),” (2) “Do you think your child’s participation in TEDDY was a good decision? (scored: 2 = a great decision, 1 = a good decision, 0 = an ok decision or bad decision)’” and (3) “Would you recommend the TEDDY study to a friend? (scored: 2 = yes, 1 = maybe, 0 = no).” Since these items were highly correlated, they were summed to create a total satisfaction score with a range of 0–6, with higher scores indicating greater satisfaction with study participation. The measure is reliable for both mothers (*α* = 0.77 on the child-age 15-month questionnaire) and fathers (*α* = 0.81) and showed evidence of stability between responses on the child-age 6-month and 15-month questionnaires (*r* = 0.66 for mothers and *r* = 0.65 for fathers). This measure has been used in previous studies with populations at-risk for T1D [10–12, 22, 23].

Father’s participation in the study is measured by completion of the father’s annual questionnaire at child-age 15 months (yes/no).

### Data analysis

ANOVA was used for continuous variables and the Chi-squared test for categorical variables when testing for univariate differences in means and proportions, respectively, between groups. Multiple linear regression was used to identify variables collected in year 1 of the TEDDY study that predicted study visit compliance during the subsequent 3 years (until child-age 4 years). Variables were added into the regression model in 5 different blocks (sociodemographic, mother’s lifestyle behaviors, post-partum depression, mother’s reactions to the child’s T1D risk, and mother’s study satisfaction/father’ participation); variables with a *p* value < 0.10 were retained in the analysis until the final model. The final model only included variables with a *p* value < 0.05. To ease the interpretation of the variables for mother’s age at the child’s birth and mother’s anxiety measured with the SAI, we divided the mother’s age by five and the SAI by its standard deviation (SD = 9.5). A follow-up analysis examined whether father study satisfaction, measured in the first year of the TEDDY study, provided an additional significant contribution to the model. Because prior studies have found that participating in TEDDY using a long-distance protocol, instead of attending a TEDDY study center, is associated with poorer study compliance [11, 24], whether or not the child was on a long-distance protocol (*n* = 284) was treated as a co-variate in all models.

All statistical analysis were conducted using SPSS version 27 (IBM SPSS Statistics for Windows, Armonk, Ny: IBM Corp).

## Results

Among the included families (*n* = 4600) in this analysis, *n* = 2784 (60.5%) completed all 11 study visits scheduled between child-age 18 and 48 months (*M* = 10.1, SD = 1.5, range 3–11). All variables used in the analysis collected before or at the child-age 15-month visit are described in Table 1. Also provided are the univariate associations of these variables to the number of study visits subsequently completed between child-age 18 and 48 months. At the univariate level, several variables were unrelated to missed visits during child-age 18–48 months: child sex, whether the mother was a single parent, whether the mother was working outside the home at child-age 9 months, and whether the mother believed something could be done to reduce the child’s T1D risk (assessed at the child-age 15-month study visit).

**Table 1:** Associations between early collected study variables and study visit compliance in the subsequent 3 years

Variable	*n* (%) or mean (SD)	Mean (SD) of number of completed visits or correlation (*r*) with number of completed visits. From 18–48 months of age	*p* value^
*Sociodemographic*			
Country			< 0.001
US	1754 (38.1)	10.11 (1.42)	
Finland	1058 (23.0)	9.92 (1.57)	
Germany	204 (4.4)	8.30 (2.26)	
Sweden	1584 (34.4)	10.49 (1.03)	
Child sex			0.814
Female	2247 (48.8)	10.11 (1.42)	
Male	2353 (51.2)	10.12 (1.78)	
First degree relative with type 1 diabetes			< 0.001
No	4196 (91.2)	10.15 (1.42)	
Yes	404 (8.8)	9.75 (1.78)	
Child ethnic minority			0.001
No	3890 (84.6)	10.16 (1.42)	
Yes	590 (12.8)	9.93 (1.56)	
Missing	120 (2.6)		
First child			0.003
No	2639 (57.4)	10.08 (1.47)	
Yes	1917 (41.7)	10.21 (1.39)	
Missing	44 (1.0)		
Parents living together			0.687
No	132 (2.9)	9.93 (1.55)	
Yes	4427 (96.2)	10.14 (1.43)	
Missing	41 (0.9)		
Mothers education			< 0.001
Higher education	2700 (58.7)	10.22 (1.38)	
Trade school	1040 (22.6)	9.90 (1.58)	
Basic primary education	831 (18.1)	10.12 (1.44)	
Missing	29 (0.6)		
Mothers age at child’s birth	4600 31.1 (4.9)	*r* = 0.06	0.005
*Maternal lifestyle variables*^*#*^			
Smokes			< 0.001
No	4221 (91.8)	10.16 (1.42)	
Yes	344 (7.5)	9.72 (1.63)	
Missing	35 (0.8)		
Works outside home			0.227
No	2921 (63.5)	10.11 (1.46)	
Yes	1622 (35.3)	10.17 (1.38)	
Missing	57 (1.2)		
Maternal postpartum depression^*^			0.081
No	4092 (89.0)	10.17 (1.41)	
Yes	357 (7.8)	9.92 (1.61)	
Missing	151 (3.3)		
*Maternal reactions to child’s type 1 diabetes risk*			
Risk perception			0.055
Underestimate	1721 (37.4)	10.19 (1.39)	
Accurate	2837 (61.7)	10.10 (1.47)	
Missing	42 (0.9)		
Anxiety (SAI)	4342 33.8 (9.4)	*r* = 0.06	0.050
Belief that child’s T1D risk can be reduced	4558 4.8 (1.7)	*r* = 0.03	0.157
*Study-related variables*			
Mother’s study satisfaction score	4556 4.7 (1.6)	*r* = 0.13	< 0.001
Father’s study satisfaction score	4363 4.2 (1.8)	*r* = 0.07	0.001
Father’s participation^+^			< 0.001
No	579 (12.6)	9.42 (1.93)	
Yes	4021 (87.4)	10.22 (1.35)	

Excluded: children autoantibody positive before 48 months

^#^Maternal lifestyle behaviors were assessed at child-age 9 months

*Postpartum depression was measured by the Edinburgh Postnatal Depression Scale at child-age 6 months; scores ≥ 13 were considered indicative of depression

^+^Defined as completing the child-age 15-month questionnaire

^*P* value are calculated for group differences for the categorical variables and for the continuous variables the correlation between the variable and the completed visits

**Fig. 1:**
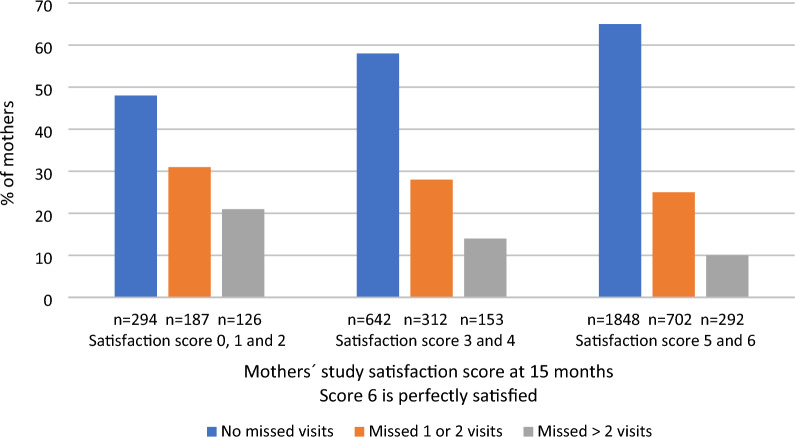
Association of Mothers’ Study Satisfaction at child-age 15 months with subsequent 3-year study visit compliance. The percentage of mothers with (1) no missed visits, (2) 1 or 2 missed visits, and (3) more than 2 missed visits from child-age 18 months to 48 months by mothers study satisfaction score at 15 months

The results of the multivariate model are provided in Table 2. Children from Sweden (*p* < 0.001), who were their mother’s first child (*p* < 0.001), who had older mothers (*p* < 0.001), whose father actively participated in the study (*p* < 0.001) and whose mothers were more satisfied with study participation during the first year of TEDDY (*p* < 0.001) completed more clinic visits in the subsequent 3 years of the study. In contrast, ethnic-minority children (*p* = 0.049), children who had mothers who smoked during their infancy (*p* < 0.001), whose mothers had high scores on the Edinburgh Postnatal Depression scale (*p* = 0.034), and whose mothers expressed higher anxiety about the child’s T1D risk during the first year of TEDDY (*p* = 0.002) completed fewer study visits in the subsequent 3 years of TEDDY. Of mothers with the highest possible study satisfaction scores at child-age 15 months (scores of 5 or 6; *n* = 2842), 65% completed all study visits between 18 and 48 months compared to 48% of mothers least satisfied with the study [scores 0–2; (*n* = 607)] (Fig. 1).

**Table 2:** Final multivariate model associations between early collected variables and subsequent study visit compliance (mothers data)

Variable	*n*	*B*	95%CI	*p* value
*Sociodemographic*				
Country				< 0.001
Sweden	1491		Reference	
US	1426	− 0.17	− 0.26, − 0.08	
Germany	185	− 1.85	− 2.07, − 1.62	
Finland	899	− 0.36	− 0.47, − 0.26	
Child ethnic minority				0.049
No	3520		Reference	
Yes	481	− 0.12	− 0.25, 0.000	
First child				< 0.001
No	2303		Reference	
Yes	1698	0.15	0.07, 0.23	
Mother’s age at child’s birth°	4001	0.09	0.04, 0.13	< 0.001
*Maternal Lifestyle variables^*				
Mother smokes				< 0.001
No	3728		Reference	
Yes	273	− 0.32	− 0.47, − 0.16	
Maternal post-partum depression^*^				0.034
No	3686		Reference	
Yes	315	− 0.15	− 0.29, − 0.01	
*Maternal reactions to child’s type 1 diabetes risk*				
Anxiety (SAI)^#^	4001	− 0.06	− 0.10, − 0.02	0.002
*Study-related variables*				
Maternal study satisfaction^¤^	4001	0.06	0.04, 0.09	< 0.001
Father’s participation^§^				< 0.001
No	303		Reference	
Yes	3698	0.60	0.46, 0.74	

Subject excluded: children islet autoantibody positive before 4 years of age. Long Distance Participation status was used as a covariate in the analysis.

°Mother’s age. In the analysis mother’s age/5 is used. ^Maternal lifestyle behaviors were assessed at child-age 9 months. *Postpartum depression was measured by the Edinburgh Postnatal Depression Scale at child-age 6 months; scores ≥ 13 were considered indicative of depression. ^#^Mothers anxiety was measured by SAI at child-age ≤ 15 months. In the analysis mothers SAI/SD is used. ^¤^Mothers study satisfaction was measured at child-age ≤ 15 months. ^§^Father’s study participation was measured at child-age 15 months

Several variables significantly related to subsequent study visit completion in the univariate analyses (Table 1) were no longer significant in the multivariate analysis: family history of T1D, maternal education, and maternal T1D risk perception accuracy (which only approached significance in the univariate model).

In a subsequent analysis, we reran the model using fathers’—instead of mothers’—data and found father’s study satisfaction at child-age 15 months also was associated with subsequent study visit compliance (*p* = 0.029; see Table 3). However, when father’s study satisfaction at child-age 15 months was added to the mothers’ model depicted in Table 2, it was not significant. The correlation between mother’s and father’s study satisfaction at child-age 15 months was *r* = 0.413. Finally, we reran the multivariate model, eliminating whether or not the child was on a long-distance protocol as a covariate; the results did not change.

**Table 3:** Final multivariate model associations between early collected variables and subsequent study visit compliance (fathers data)

Variable	*n*	*B*	95%CI	*p* value
*Sociodemographic*				
Country				< 0.001
Sweden	1492		Reference	
US	1472	− 0.19	− 0.29, − 0.10	
Germany	188	− 1.93	− 2.15, − 1.71	
Finland	909	− 0.41	− 0.51, − 0.30	
Child ethnic minority				0.009
No	3566		Reference	
Yes	495	− 0.16	− 0.29, − 0.04	
First child				< 0.001
No	2339		Reference	
Yes	1722	0.16	0.08, 0.24	
Father’s age at child’s birth	4061	0.07	0.04, 0.11	< 0.001
*Study-related variables*				
Fathers’ study satisfaction	4061	0.02	0.002, 0.05	0.029

Subject excluded: children islet autoantibodies positive before 4 years of age

Long distance participation status was used as a covariate in the analysis

°Father’s age. In the analysis fathers age/5 is used

^¤^Fathers’ study satisfaction was measured at child-age ≤ 15 months

## Discussion

No previous pediatric longitudinal screening studies have comprehensively investigated factors collected in the first year of the study that predicted study visit compliance in the subsequent 3 years of the study. Consistent with prior reports [7, 25, 26], we found the child’s ethnic-minority status was associated with poorer study visit compliance, suggesting that ethnic-minority families may need greater—or different kinds of—support to be successfully engaged in research studies.

Older mothers and mothers whose TEDDY child was their first child were more likely to complete clinic visits, while mothers who smoked were less compliant; these same variables have previously been found to be associated with study drop-out [6, 10]. Older mothers have more life experience and may be more psychologically mature and more financially stable, enabling them to better manage the demanding TEDDY research protocol. When the TEDDY child is the mother’s first child, she may feel she has sufficient time to devote to study tasks as she is not caring for additional children at the start of the TEDDY study. In contrast, mothers who smoke may be less health conscious and, therefore, more likely to complete fewer study visits or withdraw from a study.

Although prior studies have found family history of the disease was associated with study compliance [7, 11], we did not find that to be the case in the current study. This suggests that role of a family history of a disease may differ between those with a disease vs those at risk for a disease. Similarly, family history may be more or less important to compliance with certain aspects of a study protocol. For example, parents who are already living with diabetes may view an Oral Glucose Tolerance Test as less important—since they are well aware of the signs and symptoms of T1D—compared to parents with no family history with the disease, leading to differential rates of compliance with an Oral Glucose Tolerance Test study protocol. In contrast, study visits to monitor a child for the development of islet autoantibodies may be equally important to both groups, resulting in no differences in study visit compliance between those with and without a family history of the disease.

We found that Swedish families were more likely to follow the study protocol and complete their study visits compared to families from the US, Finland, and Germany. Although all sites follow the same protocol, there are important operational differences. For example, Sweden assigns a single staff person to each TEDDY child, emphasizing staff continuity, while children in US sites may see a different study staff person at different clinical visits. Another explanation may be the different length of the maternal leave in Europe compared to US; it’s more common in the European countries to stay home during the first year of the child’s life and it may influence the family’s ability to manage the demanding study protocol. Although these site differences were not the focus of this study, future studies may benefit from greater consideration of staffing practices and other contextual factors associated with study visits.

A systematic literature review found mixed findings with regard to maternal depression; some studies found mother’s level of depression was associated with study withdrawal, but others did not find that association [27]. In TEDDY, maternal post-partum depression was not associated with drop-out [10], but was associated with lower study visit compliance up to child-age 48 months. A previous publication noted that mothers with mild to moderate levels of depression stayed in the study but were more likely to need additional time to complete the study protocol [2]. Maternal depression may not always lead to study drop-out, although these mothers may be more likely to miss study visits and need more support to be able to manage a demanding research protocol.

Mother’s anxiety about the child’s T1D risk was also associated with lower study visit compliance. Similar to post-partum depression, maternal anxiety about the child’s T1D may not always be associated with study dropout [10] but is associated with lower study visit compliance [28]. Anxious mothers, similar to mothers with post-partum depression, may stay in the study but may be more likely to miss study visits, perhaps in an effort to avoid increased anxiety associated with any TEDDY visit.

Father’s participation in the TEDDY study has been found to be associated with study retention; families whose fathers did not complete his study questionnaire were more likely to withdraw from the study [6, 10]. In the current study, 87% of the fathers completed the questionnaire at child-age 15 months and father participation was found to be associated with study visit compliance in the subsequent 3 years. We can only speculate that in a family, where both parents are actively participating, the burden can be shared or at least the mother feels emotionally supported by the father in her efforts to comply with the TEDDY visit schedule.

The TEDDY study previously reported that maternal study satisfaction in the first year of study participation predicted study drop-out in the subsequent 2–3 years; more dissatisfied mothers were more likely to drop out [10]. In the current study, we found mother study satisfaction at the end of the first year of the TEDDY study predicted study visit compliance from child-age 18–48 months; more satisfied mothers after 1 year in the study are more likely to complete more study visits in the subsequent 3 years of the study. We also found that father satisfaction after 1 year in the study was also related to study visit compliance, with more satisfied fathers after 1 year in TEDDY having children who completed more clinical visits in the subsequent 3 years. However, father study satisfaction did not contribute above and beyond mother satisfaction in our multivariate model, perhaps because the two are moderately correlated. In TEDDY, study satisfaction has been associated with both site-related factors (country, staff consistency) and parental characteristics (accuracy of the parent’s perception of the child T1D risk and beliefs that something can be done to prevent the disease in the child) [12]. Since maternal study satisfaction has been associated with TEDDY drop-out [10], Oral Glucose Tolerant Test compliance [10, 11], and study visit compliance, this potentially modifiable variable may be an important variable to consider in future study design.

Our findings could be used to improve study visit compliance in several ways. Variables collected early in a study that are associated with subsequent study visit compliance could be used to create a risk score to identify the subgroup of participants at greatest risk for poor visit compliance. These families would then have additional resources devoted to them to help them meet the study protocol. Previously, TEDDY successfully used this approach to identify families at risk for early drop-out; by targeting these families with additional staff attention, these at-risk families’ retention rates improved to those of families not at-risk [6].

A second approach would be to target modifiable variables shown to be associated with study visit compliance. We found two modifiable variables associated with study visit compliance: mother’s anxiety about the child’s T1D risk, and parent’s study satisfaction. Helping highly anxious mothers successfully cope with their anxiety and efforts to ensure high parent study satisfaction may in turn increase study visit compliance.

Study limitations include our focus on young children up to 4 years of age, at risk for T1D, followed with a very demanding research protocol. Many of the participating mothers were highly educated and most mothers were living with a spouse or partner. Factors associated with study visit compliance in this population may differ from other populations, including older children, children with no risk for T1D or who face different health challenges, or families with less educated parents. The TEDDY study protocol is demanding and several of the collected variables are unique to this study making our findings perhaps less relevant for studies with less demanding research protocols. The small effect size of some of the variables is also a limitation. Although the findings do not permit prediction of study visit compliance at the individual level, they do tell us which variables—collected early in a longitudinal study—are most important in discriminating those groups of participants who will be more or less compliant with study visits in the future. This information could be useful in the design of future pediatric longitudinal studies.

Since we purposely excluded families, whose children became positive for one or more T1D-related autoantibodies, our findings cannot be generalized to this important subgroup. Mothers of these children experience significant anxiety in response to their child’s positive autoantibody status [18]. We are in the process of examining how this change in the child’s T1D risk status also impacts study compliance.

## Conclusion

Sociodemographic variables, maternal characteristics—including study satisfaction—and father participation in the first year of a longitudinal study were found to be associated with subsequent study visit compliance in children genetically at-risk for T1D followed for 4 years. This information can contribute to future strategies designed to improve study visit compliance in longitudinal pediatric studies.

### Supplementary Information

**Additional file 1.** Additional members of the TEDDY Study Group.

## Data Availability

The data sets generated and analyzed during the current study will be made available in the NIDDK Central Repository at https://repository.niddk.nih.gov/studies/teddy.

## Abbreviations

HLA: Human leukocyte antigen
SAI: State anxiety inventory
STAI: State Trait Anxiety Inventory
TEDDY: The Environmental Determinants of Diabetes in the Young
T1D: Type 1 diabetes

## Footnotes

1 Copyright © 1968, 1977 by Charles D. Spielberger. State- Trait Anxiety Inventory for Adults requires license purchase and is a trademark of Mind Garden, Inc.

## References

[1] DeMauro SB, Bellamy SL, Fernando M, Hoffmann J, Gratton T, Schmidt B (2019). Patient, family, and center-based factors associated with attrition in neonatal clinical research: a prospective study. Neonatology.

[2] Driscoll KA, Killian M, Johnson SB, Silverstein JH, Deeb LC (2009). Predictors of study completion and withdrawal in a randomized clinical trial of a pediatric diabetes adherence intervention. Contemp Clin Trials.

[3] Janus M, Goldberg S (1997). Factors influencing family participation in a longitudinal study: comparison of pediatric and healthy samples. J Pediatr Psychol.

[4] Bender BG, Ellison MC, Gleason M, Murphy JR, Sundstrom DA, Szefler SJ (2003). Minimizing attrition in a long-term clinical trial of pediatric asthma. Ann Allergy Asthma Immunol.

[5] Sullivan JA, Wiese AM, Boone KM, Rausch J, Keim SA (2020). To attend, or not to attend: examining caregiver intentions and study compliance in a pediatric, randomized controlled trial. Clin Trials.

[6] Johnson SB, Lee HS, Baxter J, Lernmark B, Roth R, Simell T (2011). The Environmental Determinants of Diabetes in the Young (TEDDY) study: predictors of early study withdrawal among participants with no family history of type 1 diabetes. Pediatr Diabetes.

[7] Hamstra MS, Pemberton VL, Dagincourt N, Hollenbeck-Pringle D, Trachtenberg FL, Cnota JF (2020). Recruitment, retention, and adherence in a clinical trial: The Pediatric Heart Network's Marfan Trial experience. Clin Trials.

[8] Besser REJ, Bell KJ, Couper JJ, Ziegler AG, Wherrett DK, Knip M (2022). ISPAD clinical practice consensus guidelines 2022: stages of type 1 diabetes in children and adolescents. Pediatr Diabetes.

[9] Teddy Study G (2007). The Environmental Determinants of Diabetes in the Young (TEDDY) study: study design. Pediatr Diabetes.

[10] Johnson SB, Lynch KF, Baxter J, Lernmark B, Roth R, Simell T (2016). Predicting later study withdrawal in participants active in a longitudinal birth cohort study for 1 year: The TEDDY Study. J Pediatr Psychol.

[11] Driscoll KA, Tamura R, Johnson SB, Gesualdo P, Clasen J, Smith L (2021). Adherence to oral glucose tolerance testing in children in stage 1 of type 1 diabetes: The TEDDY study. Pediatr Diabetes.

[12] Melin J, Lynch KF, Lundgren M, Aronsson CA, Larsson HE, Johnson SB (2022). Is staff consistency important to parents' satisfaction in a longitudinal study of children at risk for type 1 diabetes: the TEDDY study. BMC Endocr Disord.

[13] Hagopian WA, Erlich H, Lernmark A, Rewers M, Ziegler AG, Simell O (2011). The Environmental Determinants of Diabetes in the Young (TEDDY): genetic criteria and international diabetes risk screening of 421 000 infants. Pediatr Diabetes.

[14] Cox JL, Holden JM, Sagovsky R (1987). Detection of postnatal depression. Development of the 10-item Edinburgh Postnatal Depression Scale. Br J Psychiatry.

[15] Hood KK, Johnson SB, Baughcum AE, She JX, Schatz DA (2006). Maternal understanding of infant diabetes risk: differential effects of maternal anxiety and depression. Genet Med.

[16] Spielberger CDGR, Lushene R (1970). Test manual for the state-trait anxiety inventory.

[17] Marteau TM, Bekker H (1992). The development of a six-item short-form of the state scale of the Spielberger State-Trait Anxiety Inventory (STAI). Br J Clin Psychol.

[18] Johnson SB, Lynch KF, Roth R, Schatz D, Group TS (2017). My child is islet autoantibody positive: impact on parental anxiety. Diabetes Care.

[19] Melin J, Maziarz M, Andrén Aronsson C, Lundgren M, Elding LH (2020). Parental anxiety after 5 years of participation in a longitudinal study of children at high risk of type 1 diabetes. Pediatr Diabetes.

[20] Swartling U, Lynch K, Smith L, Johnson SB, Group TS (2016). Parental estimation of their child's increased type 1 diabetes risk during the first 2 years of participation in an international observational study: results from the TEDDY study. J Empir Res Hum Res Ethics.

[21] Smith LB, Lynch KF, Baxter J, Lernmark B, Roth R, Simell T (2014). Factors associated with maternal-reported actions to prevent type 1 diabetes in the first year of the TEDDY study. Diabetes Care.

[22] Johnson SB, Baughcum AE, Hood K, Rafkin-Mervis LE, Schatz DA, Group DPTS (2007). Participant and parent experiences in the parenteral insulin arm of the diabetes prevention trial for type 1 diabetes. Diabetes Care.

[23] Johnson SB, Baughcum AE, Rafkin-Mervis LE, Schatz DA, Group DPTS (2009). Participant and parent experiences in the oral insulin study of the diabetes prevention trial for type 1 diabetes. Pediatr Diabetes.

[24] Yang J, Lynch KF, Uusitalo UM, Foterek K, Hummel S, Silvis K (2016). Factors associated with longitudinal food record compliance in a paediatric cohort study. Public Health Nutr.

[25] Baxter J, Vehik K, Johnson SB, Lernmark B, Roth R, Simell T (2012). Differences in recruitment and early retention among ethnic minority participants in a large pediatric cohort: the TEDDY Study. Contemp Clin Trials.

[26] Williams NA, Coday M, Somes G, Tylavsky FA, Richey PA, Hare M (2010). Risk factors for poor attendance in a family-based pediatric obesity intervention program for young children. J Dev Behav Pediatr.

[27] Robinson L, Adair P, Coffey M, Harris R, Burnside G (2016). Identifying the participant characteristics that predict recruitment and retention of participants to randomised controlled trials involving children: a systematic review. Trials.

[28] Roth R, Lynch K, Lernmark B, Baxter J, Simell T, Smith L (2015). Maternal anxiety about a child's diabetes risk in the TEDDY study: the potential role of life stress, postpartum depression, and risk perception. Pediatr Diabetes.

