# Supplementary material for: Factors assessed in the first year of a longitudinal study predict subsequent study visit compliance: the TEDDY study
Source: Eur J Med Res. 2023 Dec 15;28:592. doi: 10.1186/s40001-023-01563-z (PMC10724932; doi:10.1186/s40001-023-01563-z)
Supplement: Supplementary file 1 — Additional file 1. Additional members of the TEDDY Study Group. [file 40001_2023_1563_MOESM1_ESM.docx]

**Acknowledgment:**

**The TEDDY study group:**

**Colorado Clinical Center:** Marian Rewers, M.D., Ph.D., PI, Kimberly Bautista^1^, Judith Baxter, Daniel Felipe-Morales, Brigitte I. Frohnert, M.D., Ph.D., Marisa Stahl, M.D., Isabel Flores Garcia, Patricia Gesualdo, Sierra Hays, Michelle Hoffman, Randi Johnson, Ph.D., Rachel Karban, Edwin Liu, M.D., Leila Loaiza, Jill Norris, Ph.D. Holly O’Donnell, Ph.D., Loana Thorndahl, Andrea Steck, M.D., Kathleen Waugh.

**Finland Clinical Center:** Jorma Toppari, M.D., Ph.D., PI, Olli G. Simell, M.D., Ph.D., Annika Adamsson, Ph.D., Suvi Ahonen, Mari Åkerlund, Sirpa Anttila, Leena Hakola, Anne Hekkala, M.D., Tiia Honkanen, Teija Hurskainen , Heikki Hyöty, M.D., Ph.D., Jorma Ilonen, M.D., Ph.D., Saori Itoshima, M.D., Minna Jokipolvi, Sanna Jokipuu, Taru Karjalainen, Leena Karlsson, Jukka Kero, M.D., Ph.D., Marika Korpela, Jaakko J. Koskenniemi M.D., Ph.D., Miia Kähönen, Mikael Knip, M.D., Ph.D., Minna-Liisa Koivikko, Katja Kokkonen, Merja Koskinen, Mirva Koreasalo, Kalle Kurppa, M.D., Ph.D., Salla Kuusela, M.D., Jarita Kytölä, Mia Laakso, Jutta Laiho, Ph.D., Tiina Latva-aho, Siiri Leisku, Laura Leppänen, Katri Lindfors, Ph.D., Maria Lönnrot, M.D., Ph.D., Elina Mäntymäki, Markus Mattila, Maija Miettinen, Tiina Niininen, Sari Niinistö, Noora Nurminen, Sami Oikarinen, Ph.D., Hanna-Leena Oinas, Paula Ollikainen, Zhian Othmani, Sirpa Pohjola, Solja Raja-Hanhela, Jenna Rautanen, Anne Riikonen, Minna Romo, Juulia Rönkä, Nelli Rönkä, Satu Simell, M.D., Ph.D., Aino Tihinen, Päivi Tossavainen, M.D., Mari Vähä-Mäkilä, Eeva Varjonen, Riitta Veijola, M.D., Ph.D., Irene Viinikangas, Silja Vilmi, Suvi M. Virtanen, M.D., Ph.D..

**Georgia/Florida Clinical Center:** Richard McIndoe, Ph.D., PI, Desmond Schatz, M.D., Diane Hopkins, Michael Haller, M.D., Melissa Gardiner, Ashok Sharma, Ph.D., Laura Jacobsen, M.D., Percy Gordon, Jennifer Hosford, Sharon Maina, Chelsea Salmon.

**Germany Clinical Center:** Anette G. Ziegler, M.D., PI, Ezio Bonifacio Ph.D., Cigdem Gezginci, Willi Grätz, Anja Heublein, Sandra Hummel, Ph.D., Annette Knopff, Sibylle Koletzko, M.D., Claudia Ramminger, Roswith Roth, Ph.D., Jennifer Schmidt, Marlon Scholz, Joanna Stock, Katharina Warncke, M.D., Lorena Wendel, Christiane Winkler, Ph.D.

**Sweden Clinical Center:** Åke Lernmark, Ph.D., PI, Daniel Agardh, M.D., Ph.D., Carin Andrén Aronsson, Ph.D., Rasmus Bennet, Corrado Cilio, Ph.D., M.D., Susanne Dahlberg, Malin Goldman Tsubarah, Emelie Ericson-Hallström, Lina Fransson, Emina Halilovic, Susanne Hyberg, Berglind Jonsdottir, M.D., Ph.D., Naghmeh Karimi, Helena Elding Larsson, M.D., Ph.D., Marielle Lindström, Markus Lundgren, M.D., Ph.D., Marlena Maziarz, Ph.D., Jessica Melin, Kobra Rahmati, Anita Ramelius, Falastin Salami, Ph.D., Anette Sjöberg, Evelyn Tekum Amboh, Carina Törn, Ph.D., Ulrika Ulvenhag, Terese Wiktorsson, Åsa Wimar.

**Washington Clinical Center:** William A. Hagopian, M.D., Ph.D., PI, Michael Killian, Claire Cowen Crouch, Jennifer Skidmore, Trevor Bender, Megan Llewellyn, Cody McCall, Arlene Meyer, Jocelyn Meyer, Denise Mulenga, Nole Powell, Jared Radtke, Shreya Roy, Preston Tucker.

**Pennsylvania Satellite Center:** Dorothy Becker, M.D., Margaret Franciscus, MaryEllen Dalmagro-Elias Smith, Ashi Daftary, M.D., Mary Beth Klein, Chrystal Yates.

**Data Coordinating Center:** Jeffrey P. Krischer, Ph.D., PI, Rajesh Adusumali, Sarah Austin-Gonzalez, Maryouri Avendano, Sandra Baethke, Brant Burkhardt, Ph.D., Martha Butterworth, Nicholas Cadigan, Joanna Clasen, Ph.D., Kevin Counts, Laura Gandolfo, Jennifer Garmeson, Veena Gowda, Christina Karges, Shu Liu, Xiang Liu, Ph.D., Kristian Lynch, Ph.D. , Jamie Malloy, Lazarus Mramba, Ph.D., Cristina McCarthy, Jose Moreno, Hemang M. Parikh, Ph.D., Cassandra Remedios, Chris Shaffer, Susan Smith, Noah Sulman, Ph.D., Roy Tamura, Ph.D., Dena Tewey, Henri Thuma, Michael Toth, Ulla Uusitalo, Ph.D., Kendra Vehik, Ph.D., Ponni Vijayakandipan, Melissa Wroble, Jimin Yang, Ph.D., R.D., Kenneth Young, Ph.D. *Past staff: Michael Abbondondolo, Lori Ballard, Rasheedah Brown, David Cuthbertson, Stephen Dankyi, Christopher Eberhard, Steven Fiske, David Hadley, Ph.D., Kathleen Heyman, Belinda Hsiao, Francisco Perez Laras, Hye-Seung Lee, Ph.D., Qian Li, Ph.D., Colleen Maguire, Wendy McLeod, Aubrie Merrell, Steven Meulemans, Ryan Quigley, Laura Smith, Ph.D.*

**Project scientist:** Beena Akolkar, Ph.D. National Institutes of Diabetes and Digestive and Kidney Diseases, Bethesda, MD, USA.

**Other contributors:** Thomas Briese, Ph.D, Todd Brusko, Ph.D., Teresa Buckner, Ph.D., Eoin McKinney, Ph.D., Tomi Pastinen, M.D., Ph.D., Steffen Ullitz Thorsen, M.D., Ph.D., Eric Triplett, Ph.D.
